# Mapping transcription mechanisms from multimodal genomic data

**DOI:** 10.1186/1471-2105-11-S9-S2

**Published:** 2010-10-28

**Authors:** Hsun-Hsien Chang, Michael McGeachie, Gil Alterovitz, Marco F Ramoni

**Affiliations:** 1 Children’s Hospital Informatics Program, Harvard-MIT Division of Health Sciences and Technology, Harvard Medical School, Boston, Massachusetts, USA; 2 Channing Lab, Brigham and Women’s Hospital, Boston, Massachusetts, USA

## Abstract

**Background:**

Identification of expression quantitative trait loci (eQTLs) is an emerging area in genomic study. The task requires an integrated analysis of genome-wide single nucleotide polymorphism (SNP) data and gene expression data, raising a new computational challenge due to the tremendous size of data.

**Results:**

We develop a method to identify eQTLs. The method represents eQTLs as information flux between genetic variants and transcripts. We use information theory to simultaneously interrogate SNP and gene expression data, resulting in a *Transcriptional Information Map* (TIM) which captures the network of transcriptional information that links genetic variations, gene expression and regulatory mechanisms. These maps are able to identify both cis- and trans- regulating eQTLs. The application on a dataset of leukemia patients identifies eQTLs in the regions of the *GART*, *PCP4*, *DSCAM*, and *RIPK4* genes that regulate *ADAMTS1*, a known leukemia correlate.

**Conclusions:**

The information theory approach presented in this paper is able to infer the dependence networks between SNPs and transcripts, which in turn can identify cis- and trans-eQTLs. The application of our method to the leukemia study explains how genetic variants and gene expression are linked to leukemia.

## Background

The mechanisms of gene transcription can be understood by the identification of genetic variants regulating gene expression (called *expression quantitative trait loci*, or eQTLs). Recent eQTL studies have taken a genome-wide approach to simultaneously analyze thousands of expression traits [1,2]. For example, Huang et al. [3] have used GWAS and expression data from HapMap individuals to identify several genetic variants that are associated with particular gene expressions related to pharmacogenomics. Most findings of eQTL associations are considered to be cis-associations, a term used to indicate that the genetic variant is in, or near, the gene whose expression it regulates [4-6]. Searching for cis-regulating SNPs is easier than searching for the opposite, trans-regulating SNPs, which regulate genes far from themselves, not only for the obvious reason that they are closer to the gene in question, but that this type of searching results in a lower multiple-testing correction and less type-1 errors [7]. The hundreds or thousands of common cis-acting variations that occur in humans may in turn affect the expression of thousands of other genes by affecting transcription factors, signaling molecules, RNA processing, and other processes that act in trans [8]. Indeed, recent research shows the promise of eQTL studies to elucidate the regulatory connections that feedback from metabolism to transcripts; thus the variations in enzyme loci can be among the most likely associations of eQTLs [9]. For all these reasons, methods that can identify trans-acting eQTLs are required to uncover the remaining biology of DNA-transcript interaction.

Identifying eQTLs requires both SNP and gene expression data in a single analysis to pinpoint the cis- and trans-SNPs modulating the expression levels. However, modern microarrays assay more than 100,000 SNPs and 50,000 genes in single chips, making direct inference of the causal interplay among such a large number of SNPs and genes a computationally infeasible task. To solve this problem, we look to information theory, and seek to construct a Transcriptional Information Map (TIM). RNA transcription is analogous to a communication system where the receiver (genes) obtains messages from the sender (SNPs) through a channel (transcription). Information theory has developed sophisticated mathematical tools to describe the properties of channels connecting receivers to transmitters. It is therefore not surprising that information theory has been a major analytical tool in bioinformatics since its early beginnings. Almost a decade ago, researchers at Children’s Hospital Informatics Program developed a highly successful information-theoretic method, known as relevance networks [10], to determine the similarity of gene expression profiles. Since then, information theory has been applied in virtually every aspect of bioinformatics [11]. For example, previous work has linked proteins in tissues to biolfuids via information theoretic channels [12]. Recently, information theory has started to emerge in eQTL analysis [13].

We extend this tradition by constructing a TIM. Information theory has provided a robust, principled framework to quantify the information flux which characterizes telecommunication channels [14]. Similarly, information theory can be applied on genomic data to reverse engineer the transcriptional information flow. In our TIMs, we model each SNP-gene pair as two nodes bridged by a channel, through which transcriptional information flows; this information is indicative of the strength or degree of gene regulation by the SNP. Analyzing the TIM can cluster SNPs and genes into groups, where dependence exists within groups but not across groups. Thus, causal analysis can be performed only within individual groups, resulting in the reconstruction of regulatory networks. We demonstrate how our approach can study transcription mechanisms in pediatric acute lymphoblastic leukemia (ALL).

## Results

We have constructed a transcriptional information map (TIM) of pediatric acute lymphoblastic leukemia (ALL), whose data was obtained from the Gene Expression Omnibus (GSE10792) [15]. In this data, 29 patients were genotyped at 100,000 SNPs using Affymetrix Human Mapping 100K Set microarrays, and the expression patterns of 50,000 genes were profiled using Affymetrix HG-U133 Plus 2.0 platforms.

### TIM of pediatric ALL

**Figure 1:**
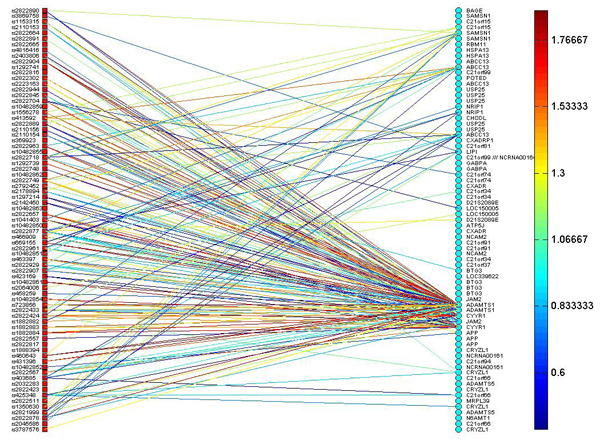
**The TIM mapping SNPs on chromosome 21q11 and genes on chromosome 21q11-q22.** The red squares denote SNPs, and blue circles denote genes. This map displays existing transcriptional channels, represented by the straight lines. The color of each line represents the signal strength of each channel as measured by mutual information.

The transcriptional information of SNP-gene pairs was quantified by mutual information. To account for noise in the data, we used a permutation test to determine the noise level, and found that a mutual information score of 0.4 or below in the ALL data could be attributed to noise. Therefore, we consider a transcriptional channel to exist between a SNP and a gene when their mutual information is above 0.4. Figure 1 shows a portion of the TIM between SNPs on chromosome 21q11 and genes on chromosome 21q11-q22; in the figure, the red squares denote SNPs, and blue circles denote genes. The map displays existing transcriptional channels, represented by the straight lines, where the color of each line represents the signal strength of each channel as mutual information**.**

### Cis/trans regulatory analysis

The TIM in Figure 1 is a tool to identify cis- and trans-eQTLs. From the map, we can trace which SNPs are linked to the genes of our interests, resulting in the recognition of candidate genomic locations whose genotypes significantly affect expression of the genes. For example, the TIM shows multiple eQTLs of *JAM2* (junctional adhesion molecule 2) which was reported to be associated with leukemia in copy number variation studies [18]. Our analysis indicates that the structural genetic variations can induce changes in expression of *JAM2,* which in turn may be a source of leukemia pathogenesis. Another gene indicated by our TIM is *ADAMTS1*, which encodes a member of the *ADAMTS* (a disintegrin and metalloproteinase with thrombospondin motif) protein family. It is located on chromosome 21q21.3, and its activation has been linked to cardiovascular disease. A study has recently found up-regulation of *ADAMTS1* in pediatric ALL samples [16], most likely due to methylation of *ADAMTS1*[17]. With reference to the TIM shown in Figure 1, it is not surprising that *ADAMTS1* has a number of cis-eQTLs on chromosome 21q21, confirming that its regulatory mechanisms are due to SNPs residing in its neighborhood [17]. However, the trans-genomic regulation of this gene by SNPs has not been previously investigated. The TIM further shows that gene *ADAMTS1* also has strong association with SNPs on cytobands q11 and q22 in chromosome 21, in addition to its known association with SNPs on cytoband q21. We have also found multiple SNPs on q11 and q22 with strong linkage to *ADAMTS1* – these indicate that the q11 and q22 regions of chromosome 21 are candidate trans-eQTLs.

A number of genes exist in the same cluster of *ADAMTS1*, including, for example, *GART*, *PCP4*, *DSCAM*, and *RIPK4*. All of these genes have similar cis- and trans-loci; they share 70% of linked SNPs in common. In this cluster, *ADAMTS1* and *GART* are known cancer biomarkers in ALL [16,19]. The involvement of *PCP4* in osteogenesis explains that abnormal bone marrow production leads to leukemia [20]. *DSCAM* and *RIPK4* have known relations to Down’s syndrome [21]; since the association of Down's syndrome and leukemia has been documented for over 70 years [22], it is not surprising that *DSCAM* and *RIPK4* are also grouped in this cluster.

### Causal regulatory analysis

**Figure 2:**
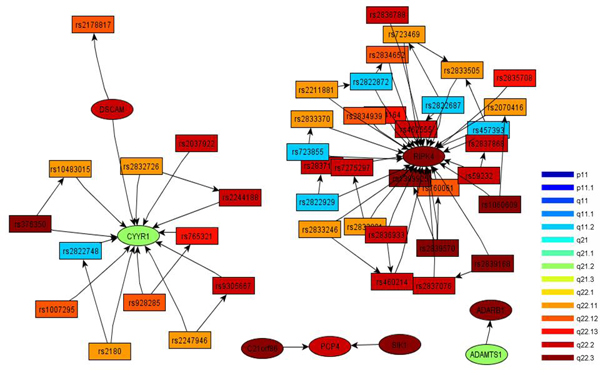
**Four cis/trans subnetworks abstracted from the TIM containing *ADAMTS1*.** These networks explain that there are 4 crucial cis and trans regulatory mechanisms centered at *RIPK4*, *CYYR1*, *PCP4*, and *ADAMTS1*. Colors encode the cytobands of the SNPs and genes.

Figure 2 displays the causal networks computed from the cluster containing the gene *ADAMTS1*. These networks explain cis-trans regulatory mechanisms. For example, *RIPK4* is a gene located at cytoband q22.3, but there are 5 distant SNPs in q11.2 (shown as blue in the figure) affecting its expression. *CYYR1*, located at chromosome 21q21.1, is a recently discovered gene [23]. It is know that it encodes a cysteine and tyrosine-rich protein, but its functional role is still under investigation, although a recent study found a correlation with neuroendocrine tumors [24]. Using our cluster analysis and abstract network analysis [25], we can infer that *CYYR1* is modulated by a number of SNPs across the q arm of chromosome 21. Furthermore, the interplay between *CYYR1* and *DSCAM* leads us to the hypothesis that *CYYR1* affects leukemia through Down’s syndrome.

## Discussion

We have presented a general framework, based on the well-established mathematics of information theory, to create a map of the relationships linking genetic variations to gene expression and regulation. We applied our methods to the analysis of genotype and gene expression for eQTL identification, where we found several established and putative eQTLs in leukemia cells. Our results have been based on a partial Transcription Information Map, while we anticipate the full TIM of leukemia will enable the identification of many new eQTLs, and subsequent experiments to test causal connections between genetic variants and gene expression and regulation.

However, TIMs need not be limited to SNP and gene expression data; other genetic data modalities are equally appropriate. For example, other genetic datasets may contain copy number variation, DNA methylations, microRNAs, or exon splicing information. With sufficient computation power, particularly parallel architectures, whole genome transcription maps can be constructed, from datasets such as the Mouse Phenome Project (http://phenome.jax.org/pub-cgi/phenome/mpdcgi). We expect that TIMs of various organisms, cells, or tissues will reveal new gene regulation mechanisms and foster the discovery and understanding of new molecular processes.

## Conclusions

This paper presents an information theory approach to infer cis- and trans-eQTLs from SNP and gene expression microarrays. Our method develops a mutual information formula between discrete and continuous variables. The mutual information captures transcriptional information flux between SNPs and genes, resulting in transcriptional information maps (TIMs). Further analyses of TIMs include grouping SNPs and genes into similar clusters, inferring causal regulation within groups, and abstracting meaningful biological networks. The application of our method on a pediatric leukemia study shows how the TIM helps to find cis- and trans-eQTLs and to extract modulation patters between SNPs and genes.

## Methods

Information theory provides a principled mathematical tool to quantify the amount of information flowing through a channel connecting a pair of nodes that are modeled by random variables. To process multimodal genomic data, we model SNPs by discrete random variables, and describe the gene expression levels by continuous random variables. A channel in the transcriptional mapping indicates that there is a transcription mechanism between the two linked nodes. The flux of transcriptional information between the two nodes is measured by mutual information. This evaluates the degree of their mutual dependence, e.g., revealing how likely it is that a gene is regulated by a SNP; a measure that includes statistical noise and microarray error.

### Computations of transcriptional information

We consider the information between a SNP-gene pair. Since the expressions are described by continuous variables, the common way of expression processing is to quantize them. However, when the sizes of quantization bins are large, the results may deviate from the true mutual information values. In contrast, when the bin sizes are small the computational time increases significantly. We avoid these quantization problems by deriving a closed-form of the mutual information between discrete and continuous variables. Let *X_j_* be a discrete variable modeling a SNP with probability mass function *p(x_j_)*, and *Y_m_* be a continuous variable modeling the expression of a gene with probability density function *f*(*y_m_*). We begin by discretizing *Y_m_* as *Y_m_*^Δ^ with bins of size Δ. Then, the mutual information (denoted by *MI*) between a discrete genetic variable *X_j_* and continuous transcript variable *Y_m_* is approximated by a discrete case:

 (1)

where *H*(*X_j_*) denotes the entropy of *X_j_*, and *x_jk_* denotes the *k*-th configuration of *X_j_*. When taking an infinitesimal bin size, the mutual information *MI* becomes:

 (2)

When *Y_m_* is a log-normal variable, it has a closed-form, [14],

 (3)

where *σ^2^_m_* denotes the variance of *Y_m_*. Similarly, its entropy conditional on *X_j_* = *x_jk_* is

 (4)

where *σ^2^_mjk_* denotes the variance of *Y_m_* conditional on *x_jk_*. Substituting equations 3 and 4 into *MI* 2 leads to the following formula

 (5)

Note that the mean values do not play a role in the definition of the entropy of normal variables. Hence, computing *MI* merely relies on the marginal and conditional variances of *Y_m_*.

**Figure 3:**
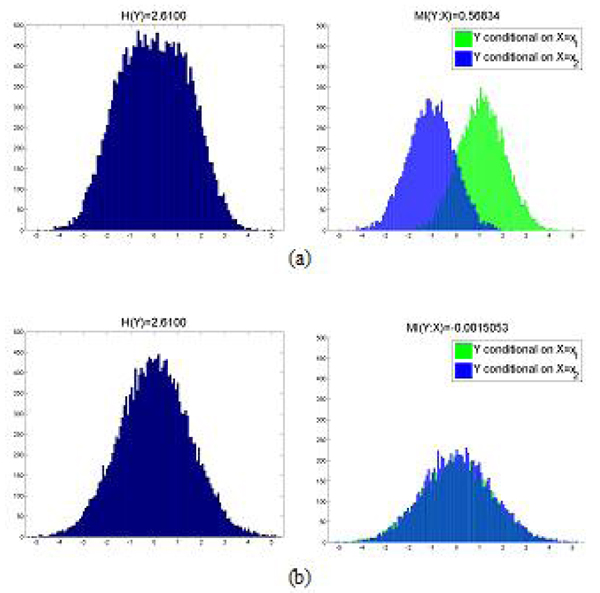
**Illustration of mutual information between discrete and continuous variables.** (a) The expression level of gene *Y* is modulated by a SNP *X*. The distribution of *Y* alone is a Gaussian with entropy *H(Y)=*2.61. When conditional on SNP *X*, the gene *Y* is a bimodal Gaussian whose mutual information with SNP *X* is *H(Y:X)*=0.57. (b) The gene *Y* and SNP *X* are independent. Although gene *Y* follows a Gaussian distribution and its entropy is the same as the entropy in (a), its distribution conditional on SNP *X* remains a unimodal Gaussian and its mutual information with SNP *X* is *H(Y:X)*=0.

We provide an example to illustrate mutual information between continuous and discrete variables. Figure 3(a) shows an example where expression level of gene *Y* is modulated by a SNP *X*. The distribution of *Y* alone is a Gaussian with entropy *H(Y)=*2.61. When conditional on SNP *X*, the gene *Y* is a bimodal Gaussian whose mutual information with SNP *X* is *H(Y:X)*=0.57. In contrast, Figure 3(b) shows the other example where gene *Y* and SNP *X* are independent. Although gene *Y* follows a Gaussian distribution and its entropy is the same as the preceding example, its distribution conditional on SNP *X* remains unimodal and its mutual information with SNP *X* is *H(Y:X)*=0.

### Identification of system noise

In a noiseless environment, transcriptional relations exist between SNP-gene pairs with mutual information greater than zero. When microarray noise and error take place, the noise level ε needs to be derived from the available data, and the mutual information above ε is deemed statistically significant. We determined the noise level ε using permutation test [10]. Ideally, we would randomly permute all the SNP and gene expression data to compute mutual information of all SNP-gene pairs, and then repeat this procedure multiple times to identify ε. However, a huge number of SNPs and genes make this impractical with the full dataset. We surmount this difficulty by sampling one tenth of SNPs and one tenth of genes and running the permutation test with a limit of 30 random permutations.

### Parallelization of TIM computation

**Figure 4:**
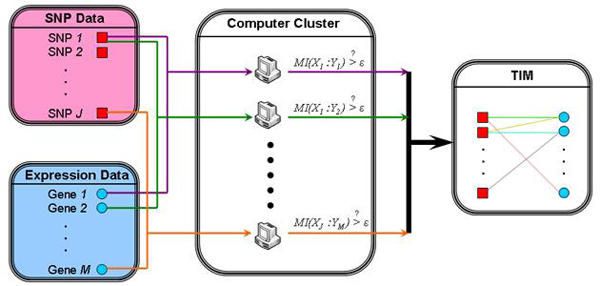
**The distributed computing system to construct a TIM from a set of SNP and gene expression data.** Each computer in the cluster is responsible for calculating a portion of the TIM. Once the mutual information of a SNP-gene pair is computed, it is immediately used to determine if the pair is independent, and the mutual information is only recorded for dependent pairs. Finally, consolidation of all computed mutual information values generates a complete TIM.

Modern microarray technologies can assay hundreds of thousands of SNPs or transcripts on a single chip. Computing the TIM of a tremendous number of SNPs and transcripts is a time consuming task. We can utilize parallel computing to enhance computational efficiency. The computations of mutual information for a pair of values described in equation (5) do not rely on other variables. Hence, we can distribute the computations of mutual information over any number of computers. Figure 4 illustrates the distribution of the TIM computation task from a set of SNP and gene expression data. The TIM can be represented mathematically by a mutual information matrix. Each computer in the cluster is responsible for calculating a portion of the matrix elements. Once an element is computed, it is immediately used to determine if the pair of nodes is independent, and the mutual information is only recorded for dependent pairs. Finally, consolidation of all computed mutual information values results in a complete TIM.

### Clustering

Visualizing and understanding results from expression studies of the entire genome at once can be overwhelming. As a result, many researchers have used cluster analysis to distill the genomic data down to a more comprehensible level [26]. There are two main elements to any clustering strategy: (i) a similarity measure that can be used to determine which genes are most related to each other, and (ii) a procedure for joining similar genes into one single gene group, or *cluster*, that summarizes and preserves the statistical properties and information interactions of the constituent genes, while at the same time reducing the size of the network.

In the TIM, linked SNP-gene pairs behave dependently, so we can use this statistical dependency as a similarity measure for cluster analysis. Within the TIM, a SNP linked by noiseless information channels to several transcripts provides evidence that these genes possess similar expression profiles. Conversely, a set of SNPs linked to the same gene share a similar genotypic pattern. Furthermore, a path (i.e., a sequence of linked SNPs and genes) in the TIM indicates that these linked nodes have dependent probability distributions, so the SNPs and genes on this path should belong to the same cluster. We can regard this cluster as the domain of a message passed from a SNP to a gene, and from the gene to another SNP again, in repeated relays. When there is a smooth channel, the message should arrive from the sender in perfect order; when the channel is clogged, the message may be misinterpreted or totally missing. In other words, SNPs and genes linked by transcriptional channels are highly likely to be involved in the same transcriptional mechanism. Isolated groups of linked SNPs and genes are considered clusters.

### Inference of causality

We carry out the inference of transcriptional interactions using a Bayesian networks method which can handle mixed types of random variables [27,28]. In a TIM, models containing clusters with no channels connecting each other signify independence between clusters, implying that the causal relations exist only *within* clusters but not *across* clusters. Hence, we can transform the TIM into a causal network by learning the optimal Bayesian networks within individual clusters. In our Bayesian network model, the causality between SNPs and genes always leads from SNPs to genes, but the complete causal inference is still complicated. First, the mapping of transcriptional information computes the (in)dependencies between SNP-gene pairs. For a disconnected pair in the TIM, there must be no causal link in the Bayesian network. For a connected pair, the causal relationship between the SNP and the gene is not necessarily from this SNP to the gene, the causal link might be through other SNPs and/or genes. Thus, the clustered TIM provides constraints on the optimal Bayesian network, leading to great gains in computational efficiency.

### Network abstraction

A cluster can contain a large number of SNPs and genes, leading to difficulty in providing meaningful biological interpretation. To extract useful biological information, we apply a holistic approach to finding hidden relationships of the network. In order to capture global topologic properties, topological distances between all the nodes are calculated via Dijkstra’s algorithm. The largest connected graph, referred to here as the global topologic profile, is then examined in detail. We further calculate a scree plot [29] to determine the dimensionality of the data.

Considering the first three principal components is enough to capture 60% of the variability and thus they are used to project, and thereby topologically abstract, the global topologic profile. In order to examine the biological information encoded, the topologic profile’s connectivity matrix is used to construct a new visual representation of the network. In the study of pediatric ALL, Figure 2 shows the final abstract networks.

## Competing interests

The authors declare they have no competing interests.

## Authors' contributions

HHC, MM, and GA designed the method and conducted the analysis; MFR directed the study; HHC, MM, and MFR prepared the manuscript.
